# A Rare and Unexpected Reason for Unilateral Epistaxis: Nasal Septal Schwannoma

**DOI:** 10.1155/2020/4369620

**Published:** 2020-10-09

**Authors:** Alper Yenigun, Yasin Kulaksiz, Tugce Esen Kiran, Erol Senturk, Fadlullah Aksoy, Orhan Ozturan

**Affiliations:** ^1^Bezmialem Vakif University, Faculty of Medicine, Department of Otorhinolaryngology, Fatih, Istanbul, Turkey; ^2^Bezmialem Vakif University, Faculty of Medicine, Department of Pathology, Fatih, Istanbul, Turkey

## Abstract

Nasal septal schwannoma is a rare tumor. It causes complaints such as nasal congestion, nosebleeds, and headaches. There are many diseases such as nasal polyps, antrochoanal polyp, chronic rhinosinusitis, concha bullosa, inverted papilloma, and retention cyst with schwannoma diagnosis. The diagnosis is made histopathologically, and the treatment is surgery. In this case report, we presented a male patient with septal schwannoma who had nasal obstruction for a year and reviewed the last 20 years of literature on nasal schwannoma.

## 1. Introduction

Schwannomas are benign tumors of the nerve sheath commonly associated with cranial nerve VIII. It is observed with a frequency of 25–45% among all head and neck tumors [1–4]. Only 4% of them are seen in the nose [1]. Nasal septum is one of the places where schwannoma is rarely seen in the nose [1–5]. In this study, we presented a case of a male with septal schwannoma who had suffered nasal congestion for a year. This study was conducted to examine the frequency of schwannomas among the causes of nasal obstruction. Therefore, we also reviewed last 20 years of literature about nasal schwannoma.

## 2. Case Report

**Figure 1:**
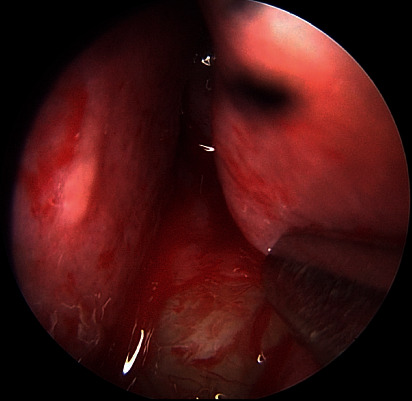
Endoscopic examination of the nose showing a large, left-sided nasal polypoid mass occluding the entire left nasal cavity.

**Figure 2:**
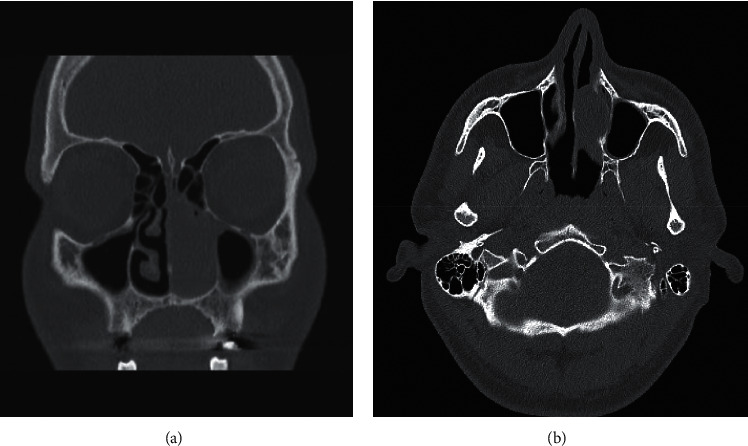
CT scan of the paranasal sinuses with contrast. The coronal and axial section shows a left anterior nasal mass.

**Figure 3:**
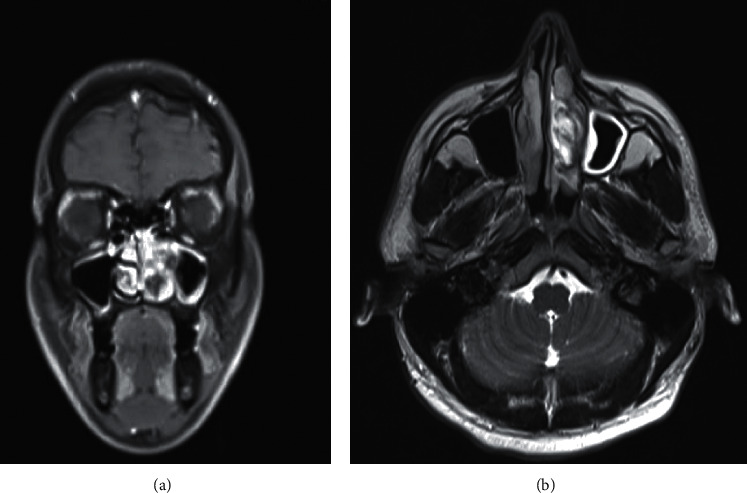
MRI scan of the paranasal sinuses with contrast. The coronal and axial section shows a left anterior heterogeneous hypointense nasal mass.

**Figure 4:**
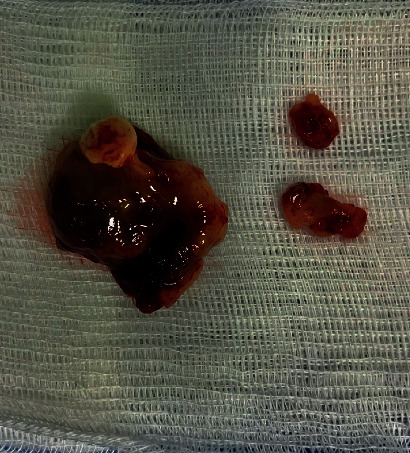
Gross image of the mass revealed multiple fragments of soft tan-gray tissue.

**Figure 5:**
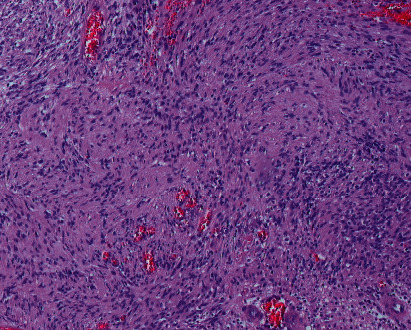
Histopathological examination showing high cellular density and a palisading pattern of the tumor cells (×40 power, H&E-stained microscopic slide picture).

**Figure 6:**
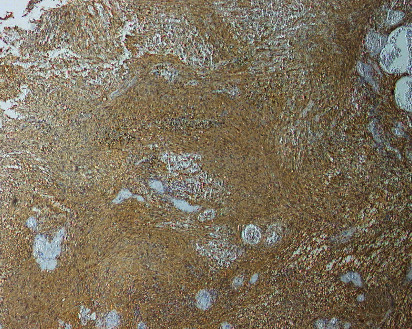
Immunohistochemistry showing tumor cells that are strongly positive for S-100.

A 28-year-old male patient was admitted to our clinic with progressive nasal congestion and recurrent epistaxis. In rigid endoscopy, there was a polypoid mass originating from the septum with smooth surface and prone to bleeding. It was causing nasal obstruction on the left side of the nose (Figure 1). In paranasal sinus computed tomography (CT), approximately 16 × 40 × 35 mm-sized polypoid mass was observed on the left side, filling the nasal cavity completely (Figure 2). No extension or bone invasion to any paranasal sinuses was present. Magnetic resonance imaging (MRI) revealed a hypointense mass and heterogeneous contrast in T1 and T2 sections (Figure 3). The surgery was planned to the patient under general anesthesia. Endoscopically mucosal incision was made proximal to where the mass attaches to the septum. The procedure was continued by subperichondrial elevation. The mass was excised en bloc with the septum mucosa (Figure 4). In the histological examination of the mass, spindle cell proliferation consisting of photocellular and hypocellular alternating areas and diffuse positivity in spindle cells with S100 were observed (Figures 5 and 6). Vimentin staining was positive, though neuron-specific enolase and smooth muscle actin staining were negative. According to these observations, the mass was defined as a septum-derived schwannoma. No recurrence was observed during the patient's 1-year follow-up.

## 3. Discussion

Schwannoma is a class sheath tumor often seen in the head and neck region. While approximately 25–45% of schwannomas are seen in the head and neck region [1–4], only 4% of them are seen in the nose [6]. The case of schwannoma in the nasal septum was first reported by Betkowski et al. in 1943 [7]. Schwannoma is seen especially in the 4^th^ and 6^th^ decades, and there is no gender dominance [8]. Nasal septal schwannoma cases reported in the last 20 years are shown in Table 1. Schwannoma often originates from the posterior part of the septum in the nose and makes complaints such as nasal congestion, nosebleeds, and headaches [6]. In our case, it originated from the anterior-middle region of the septum at young age different from the other cases. The unilateral mass in the nose was observed at 22% nasal polyps, 19% antrochoanal polyp, 13% chronic rhinosinusitis, 11% concha bullosa, 6% inverted papilloma, and 6% retention cyst. More rarely, fibrous dysplasia, lymphoma, pleomorphic adenoma, and schwannoma are seen. In such a large list, only observation is not sufficient for diagnosis [9]. Paranasal CT findings are nonspecific. CT often helps with the size of the tumor and where it originates. MRI tumor is better at differentiating from inflammatory diseases and normal tissue. It also shows intracranial extension [10]. In previous studies, findings such as “target sign” and “fascicular sign,” which are specifically observed in schwannoma tumors on MRI, have been reported [11–14].

The definitive diagnosis of schwannoma tumor is made histopathologically. Schwannoma tumors are macroscopically seen as bordered and encapsulated. When stained with hemotoxylin-eosin stain and analyzed microscopically, it is divided into two patterns, Antoni A and Antoni B. Antoni A consists of spindle cells that form palisade by lining the nuclei side by side. Antoni B consists of loose myxoid stroma and a small amount of spindle cells. It is stained strongly with S-100 immunohistochemically [4, 15, 16]. In the histological examination of the mass of our patient, spindle cell proliferation consisting of photocellular and hypocellular alternating areas with S-100 and diffuse positivity in spindle cells were observed. Although neuron-specific enolase and smooth muscle actin staining were negative, vimentin staining was positive. Based on these observations, the mass was defined as septum-derived schwannoma. Treatment of nasal septal schwannoma is extensive surgical excision with a negative surgical margin. No recurrence cases were reported in literature after excision [17–29].

## Figures and Tables

**Table 1:** Nasal septal schwannoma cases reported in the last 20 years.

Author(s)	Year	Age/sex	Symptoms	Site	Treatment	Follow-up
Berlucchi et al. [4]	2000	29/M	NO, Ep	NS	Ex	7 years
Wada et al. [18]	2000	62/F	NO	NS	Ex	15 months
Wang et al. [12]	2004	55/M	NO	NS	Ex	—
Rajagopal	2005	54/F	NO	NS	Ex	6 months
Shinohara et al. [14]	2005	50/M	NO	NS	Ex	3 years
Kodama et al. [19]	2009	41/F	NO	NS	Ex	2 years
Pauna et al. [8]	2013	78/M	NO	NS	Ex	—
Gulia et al. [20]	2013	35/F	NO	Membranous NS	Ex	6 months
Cadd et al. [21]	2014	31/M	NO, Ep	NS	Ex	—
Dhingra et al. [22]	2014	28/F	NO, Ep	NS	Ex	—
Karatas [23]	2016	31/F	NO, Ep	NS	Ex	8 months
Gerritsen et al. [24]	2017	16/M	NO	NS	Ex	1 month
Gupta et al. [25]	2017	40/F	NO, Ep	NS	Ex	1 year
Valsamidis et al. [26]	2017	54/M	NO, Ep	NS	Ex	6 months
Min et al. [3]	2017	65/F	NO	NS	Ex	2 years
Devaraja et al. [27]	2018	57/M	NO	NS	Ex	—
Alrasheed et al. [28]	2019	64/F	NO, Ep	NS	Ex	3 years
Bie et al. [29]	2019	47/M	NO, Ep	NS	Ex	6 months
Our case	2020	28/M	NO, Ep	NS	Ex	1 year

M: male; F: female; NO: nasal obstruction; Ep: epistaxis; NS: nasal septum; Ex: excision.

## References

[1] Suh J. D., Ramakrishnan V. R., Zhang P. J. (2011). Diagnosis and endoscopic management of sinonasal schwannomas. *ORL*.

[2] Cakmak O., Yavuz H., Yucel T. (2003). Nasal and paranasal sinus schwannomas. *European Archives of Oto-Rhino-Laryngology*.

[3] Min H. J., Hong S. C., Kim K. S. (2017). Nasal septal schwannoma. *Journal of Craniofacial Surgery*.

[4] Berlucchi M., Piazza C., Blanzuoli L., Battaglia G., Nicolai P. (2000). Schwannoma of the nasal septum: a case report with review of the literature. *European Archives of Oto-Rhino-Laryngology*.

[5] Ata N., Koç E., Can Y., Balta H. (2016). Schwannoma originating from the nasal septum. *Journal of Craniofacial Surgery*.

[6] Mey K. H., Buchwald C., Daugaard S., Prause J. U. (2006). Sinonasal schwannoma—a clinicopathological analysis of five rare cases. *Rhinology*.

[7] Betkowski A., Wysocka-Kuźniar S., Wewiórska T. (1979). Nerwiaki przegrody nosa neurilemmoma of the nasal septum. *Otolaryngologia Polska*.

[8] Pauna H. F., De Carvalho G. M., Guimarães A. C., Maunsell R. C. K., Sakano E. (2013). Schwannoma of the nasal septum: evaluation of unilateral nasal mass. *Brazilian Journal of Otorhinolaryngology*.

[9] Habesoglu T. E., Habesoglu M., Surmeli M., Uresin T., Egeli E. (2010). Unilateral sinonasal symptoms. *Journal of Craniofacial Surgery*.

[10] Fujiyoshi F., Kajiya Y., Nakajo M. (1997). CT and MR imaging of nasoethmoid schwannoma with intracranial extension. *American Journal of Roentgenology*.

[11] Valencia M. P., Castillo M. (2008). Congenital and acquired lesions of the nasal septum: a practical guide for differential diagnosis. *Radiographics*.

[12] Wang L.-F., Tai C.-F., Ho K.-Y., Kuo W.-R., Chai C.-Y. (2004). Schwannoma of the nasal septum: a case report. *The Kaohsiung Journal of Medical Sciences*.

[13] Pagella F., Giourgos G., Matti E., Colombo A. (2009). An asymptomatic schwannoma of the nasal septum: report of a unique case. *Ear, Nose & Throat Journal*.

[14] Shinohara K., Hashimoto K., Yamashita M., Omori K. (2005). Schwannoma of the nasal septum removed with endoscopic surgery. *Otolaryngology-Head and Neck Surgery*.

[15] Donnelly M. J., al-Sader M. H., Blayney A. W. (1992). Benign nasal schwannoma. *The Journal of Laryngology & Otology*.

[16] Fine S. W., McClain S. A., Li M. (2004). Immunohistochemical staining for calretinin is useful for differentiating schwannomas from neurofibromas. *American Journal of Clinical Pathology*.

[17] Batra P. S., Luong A., Kanowitz S. J. (2010). Outcomes of minimally invasive endoscopic resection of anterior skull base neoplasms. *The Laryngoscope*.

[18] Wada A., Matsuda H., Matsuoka K., Kawano T., Furukawa S., Tsukuda M. (2001). A case of schwannoma on the nasal septum. *Auris Nasus Larynx*.

[19] Kodama S., Okamoto T., Suzuki M. (2010). Ancient schwannoma of the nasal septum associated with sphenoid sinus mucocele. *Auris Nasus Larynx*.

[20] Gulia J. S., Yadav S. S., Basur S. K., Hooda A. (2013). Schwannoma of the membranous nasal septum. *Brazilian Journal of Otorhinolaryngology*.

[21] Cadd B., Offiah C., Alusi G. (2014). A surprising cause of unilateral nasal obstruction and epistaxis: nasal septal schwannoma. *Journal of Surgical Case Reports*.

[22] Dhingra S., Bakshi J., Mohindra S. (2014). Schwannoma of the nasal septum: an unusual finding. *Ear, Nose, &amp; Throat Journal*.

[23] Karatas A., Cebi I. T., Salviz M., Kocak A., Selcuk T. (2016). Schwannoma of the nasal septum. *Egyptian Journal of Ear, Nose, Throat and Allied Sciences*.

[24] Gerritsen R., Corao D., Shah U. K. (2017). Schwannoma of the nasal septum: rare presentation and literature review. *International Journal of Pediatric Otorhinolaryngology Extra*.

[25] Gupta M., Rao N., Kour C., Kaur I. (2017). Septal schwannoma of the nose: a rare case. *Turk Otolarengoloji Arsivi*.

[26] Valsamidis K., Koutsampasopoulou I., Titelis K. (2017). Nasal septal schwannoma: an extremely rare tumor. *Acta Otorrinolaringologica*.

[27] Devaraja K., Nayak D. R., Ramaswamy B., Rao P. (2018). Nasal septal schwannoma: a rare sinonasal tumour with certain peculiarities. *BMJ Case Reports*.

[28] Alrasheed W., Almomen A., Alkhatib A. (2019). A rare case of nasal septal schwannoma: case report and literature review. *International Journal of Surgery Case Reports*.

[29] Bie X., Wang J., Sun X., Sun K., Tang Y. (2020). Combined application of endoscope and low-temperature plasma knife in the excision of nasal septal schwannoma. *Ear, Nose & Throat Journal*.

